# Knockdown of Dehydrodolichyl Diphosphate Synthase in the *Drosophila* Retina Leads to a Unique Pattern of Retinal Degeneration

**DOI:** 10.3389/fnmol.2021.693967

**Published:** 2021-07-05

**Authors:** Tal Brandwine, Reut Ifrah, Tzofia Bialistoky, Rachel Zaguri, Elisheva Rhodes-Mordov, Liliana Mizrahi-Meissonnier, Dror Sharon, Vladimir L. Katanaev, Offer Gerlitz, Baruch Minke

**Affiliations:** ^1^Department of Medical Neurobiology, Faculty of Medicine and The Edmond and Lily Safra Center for Brain Sciences (ELSC), The Hebrew University of Jerusalem, Jerusalem, Israel; ^2^Department of Developmental Biology and Cancer Research, Faculty of Medicine, Institute for Medical Research Israel-Canada (IMRIC), The Hebrew University of Jerusalem, Jerusalem, Israel; ^3^Department of Ophthalmology, Hadassah Medical Center, Faculty of Medicine, The Hebrew University of Jerusalem, Jerusalem, Israel; ^4^Department of Cell Physiology and Metabolism, Translational Research Centre in Oncohaematology, Faculty of Medicine, University of Geneva, Geneva, Switzerland; ^5^School of Biomedicine, Far Eastern Federal University, Vladivostok, Russia

**Keywords:** DHDDS, photoreceptor degeneration, RNAi, *Drosophila*, N-glycosylation

## Abstract

Dehydrodolichyl diphosphate synthase (DHDDS) is a ubiquitously expressed enzyme that catalyzes *cis*-prenyl chain elongation to produce the poly-prenyl backbone of dolichol. It appears in all tissues including the nervous system and it is a highly conserved enzyme that can be found in all animal species. Individuals who have biallelic missense mutations in the *DHDDS* gene are presented with non-syndromic retinitis pigmentosa with unknown underlying mechanism. We have used the *Drosophila* model to compromise *DHDDS* ortholog gene (*CG10778*) in order to look for cellular and molecular mechanisms that, when defective, might be responsible for this retinal disease. The Gal4/UAS system was used to suppress the expression of *CG10778* via RNAi-mediated-knockdown in various tissues. The resulting phenotypes were assessed using q-RT-PCR, transmission-electron-microscopy (TEM), electroretinogram, antibody staining and Western blot analysis. Targeted knockdown of *CG10778*-mRNA in the early embryo using the actin promoter or in the developing wings using the *nub* promoter resulted in lethality, or wings loss, respectively. Targeted expression of *CG10778*-RNAi using the *glass multiple reporter* (GMR)-Gal4 driver (GMR-DHDDS-RNAi) in the larva eye disc and pupal retina resulted in a complex phenotype: (a) TEM retinal sections revealed a unique pattern of retinal-degeneration, where photoreceptors R2 and R5 exhibited a nearly normal structure of their signaling-compartment (rhabdomere), but only at the region of the nucleus, while all other photoreceptors showed retinal degeneration at all regions. (b) Western blot analysis revealed a drastic reduction in rhodopsin levels in GMR-DHDDS-RNAi-flies and TEM sections showed an abnormal accumulation of endoplasmic reticulum (ER). To conclude, compromising DHDDS in the developing retina, while allowing formation of the retina, resulted in a unique pattern of retinal degeneration, characterized by a dramatic reduction in rhodopsin protein level and an abnormal accumulation of ER membranes in the photoreceptors cells, thus indicating that DHDDS is essential for normal retinal formation.

## Introduction

The identification of a founder mutation in the dehydrodolichyl diphosphate synthase (*DHDDS*) gene in Ashkenazi Jews (AJ) with non-syndromic retinitis pigmentosa (RP) was reported a decade ago (Zelinger et al., 2011; Züchner et al., 2011). A single-nucleotide mutation in the *DHDDS* gene c.124A > G was found in an AJ family. The p.K42E mutation affects a highly conserved region of the DHDDS protein, which is located in close proximity to a binding site of farnesyl diphosphate. This mutation has subsequently been confirmed in other similar patients and is found in 10–20% of autosomal recessive RP in AJ population (Zelinger et al., 2011). The clinical phenotype of patients who are homozygous for the p.K42E mutation is within the spectrum often described in autosomal recessive RP (Zelinger et al., 2011; Züchner et al., 2011). Patients harboring mutations in DHDDS demonstrated fundus findings at a relatively early age. Clinically, they demonstrated waxy appearance of the optic nerve head, attenuation of retinal blood vessels and retinal atrophy in the mid and far periphery combined with significant bone spicule-like pigmentation, starting already in their 20s. The atrophic changes spread into the macular area and the pigmentary changes became denser with age (Kimchi et al., 2018). Kinetic visual fields revealed reduced peripheral function in the youngest patients studied and only small central islands of vision remaining later in life. Electroretinogram responses were not detectable in most patients (Zelinger et al., 2011). In the human retina, *DHDDS* is expressed in the inner segment of photoreceptors, where dolichol biosynthesis is predicted to occur (Zelinger et al., 2011). A possible link between insufficient DHDDS activity and photoreceptor degeneration was investigated in zebrafish, in which the expression of DHDDS was knocked down by morpholino oligonucleotides injected into zebrafish one cell embryos. The results demonstrated that suppression of DHDDS expression in zebrafish led to loss of photoreceptor outer segments and visual function (Wen et al., 2014). The results thus support the hypothesis that insufficient DHDDS function can lead to retinal degeneration. However, an additional study describing a patient with a severe multisystem disease associated with DHDDS deficiency shows that RP is not the only clinical sign in cases of DHDDS deficiency (Sabry et al., 2016).

DHDDS is a highly conserved enzyme that can be found in all animal species. It catalyzes *cis*-prenyl chain elongation to produce the poly-prenyl backbone of dolichol. The human enzyme was reported to be able to complement for the lack of the orthologous enzyme in a yeast mutant strain (Endo et al., 2003). In mammals, dolichol is present in most tissues including the nervous system (Carroll et al., 1992). In eukaryotes, the endoplasmic reticulum (ER) associated cis-prenyltransferase is the first enzyme that participates in the synthesis of dolichol phosphate, which is an indispensable lipid carrier for protein N-glycosylation (Grabińska et al., 2016). Protein N-glycosylation is a fundamental biological process important for the correct folding and function of many eukaryotic glycoproteins (West, 1986). This process occurs in eukaryotes in the lumen of the ER. Dolichyl pyrophosphate plays an important role as a glycosyl carrier in the biosynthesis of the N-linked oligosaccharide chains of freshly synthesized glycoproteins (Grabińska et al., 2016). The human cis-prenyltransferase is an enzymatic complex essential for protein N-glycosylation. It is composed of two structurally and functionally distinct subunit types (Grabińska et al., 2016; Bar-El et al., 2020; Edani et al., 2020). These include the catalytically active DHDDS and the quiescent Nogo-B receptor (NgBR) subunits (Harrison et al., 2011; Bar-El et al., 2020; Edani et al., 2020). Importantly, the distal C-terminus of NgBR transverses across the interface with DHDDS, directly participating in active-site formation and functional coupling between the two subunits (Bar-El et al., 2020). The heterodimerization architecture supports the notion that although DHDDS is considered as the catalytically active subunit, both subunits are necessary for efficient dolichol synthesis. Accordingly, by forming the cis-prenyltransferase complex, DHDDS and NgBR were shown to play a crucial role in cellular dolichol synthesis (Endo et al., 2003; Harrison et al., 2011). Mutations in human cis-prenyltransferase cause severe human diseases (Grabińska et al., 2016; Hamdan et al., 2017). Interestingly, these mutations can be subdivided into mutations that directly or indirectly interfere with substrate association. These mutations result in a similar reduction in catalytic activity and are clinically associated with developmental epileptic encephalopathies (Hamdan et al., 2017; Bar-El et al., 2020). The functional consequences of disease mutations clustered around the active-site, and in combination with molecular dynamics simulations, suggested a mechanism for the human cis-prenyltransferase dysfunction in RP (Bar-El et al., 2020).

**FIGURE 1:**
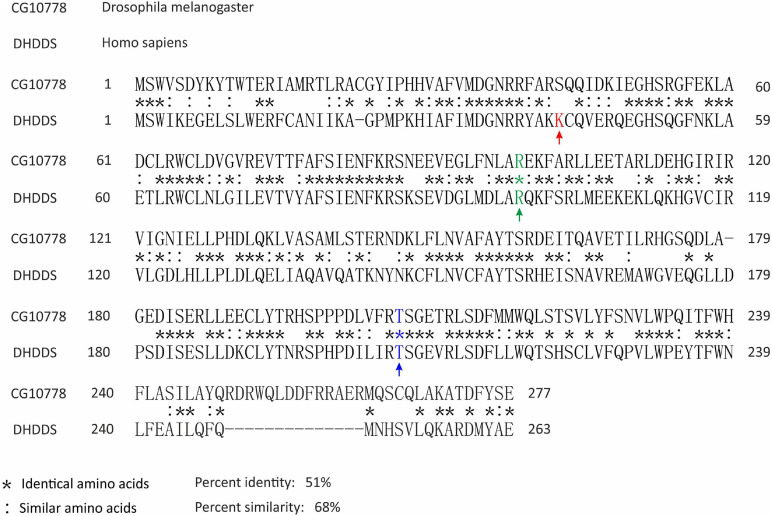
Amino acid sequence alignment of the human DHDDS and its *Drosophila* ortholog proteins. The amino acid sequences of the human DHDDS and the *Drosophila* CG10778 proteins were aligned by BLASTp. Positions with identical amino acids were labeled with (*), while positions that preserved the physico-chemical properties of the original residue were marked with (:). Marked with arrows and in color are the DHDDS sites known to be involved in non-syndromic Retinitis Pigmentosa: K42 (red), R98 (green), T206 (blue). Only R98 and T206 are identical in the *Drosophila* orthologue CG10778. NCBI accession numbers are as follows: NP_572425.1 (CG10778) and NP_995583.1 (DHDDS).

The *CG10778* gene is the only *Drosophila* ortholog of the human *DHDDS* gene (Figure 1), predicted to have DHDDS activity and polyprenyltransferase activity. CG10778 is defined in flybase as a DHDDS subunit (Larkin et al., 2021). It is predicted to have both DHDDS and polyprenyltransferase activity. It is also predicted to be involved in the polyprenol biosynthetic process, to be localized to the DHDDS complex and to the ER. Importantly, *CG10778* is the only gene predicted to have DHDDS activity as there are no paralogs of DHDDS in the *Drosophila* genome (Larkin et al., 2021), thus constituting a strong bioinformatics evidence for the function of GC10778.

N-glycosylation of the *Drosophila* rhodopsin protein, occurring in the ER, is essential for its maturation. *Drosophila* rhodopsin undergoes a complete deglycosylation before its incorporation into the photoreceptor membrane. The site of glycosylation and its role in rhodopsin synthesis were investigated by *in vitro* synthesis and *in vivo* expression of a rhodopsin mutant, whose putative N-glycosylation site (Asn-20) was replaced by isoleucine [Rh1 N20I, (O’Tousa, 1992)]. The results demonstrated that immature rhodopsin binds a single oligosaccharide chain exclusively at Asn-20 in the N-terminal extracellular domain. Furthermore, the results gave the first evidence directly indicating that preventing the attachment of the oligosaccharide chain markedly impedes rhodopsin maturation resulting in a pronounced rhodopsin reduction (Katanosaka et al., 1998). Rh1 rhodopsin elimination leads to severe shrinkage of the rhabdomeres (Kumar and Ready, 1995; Yasin et al., 2017). There is a critical developmental period in which rhodopsin plays its key role in photoreceptor morphogenesis (Kumar et al., 1997; Yasin et al., 2017). Thus, besides its function in light reception, rhodopsin plays an essential structural role during photoreceptors morphogenesis.

To gain a better understanding of the effect of DHDDS abnormalities on retinal structure and function, we used the *Drosophila* animal model and found that targeted expression of *CG10778*-RNAi using the *glass multiple reporter* (GMR)-Gal4 driver, while allowing retinal formation, resulted in a unique pattern of retinal degeneration.

## Materials and Methods

### Fly Stocks

**FIGURE 2:**
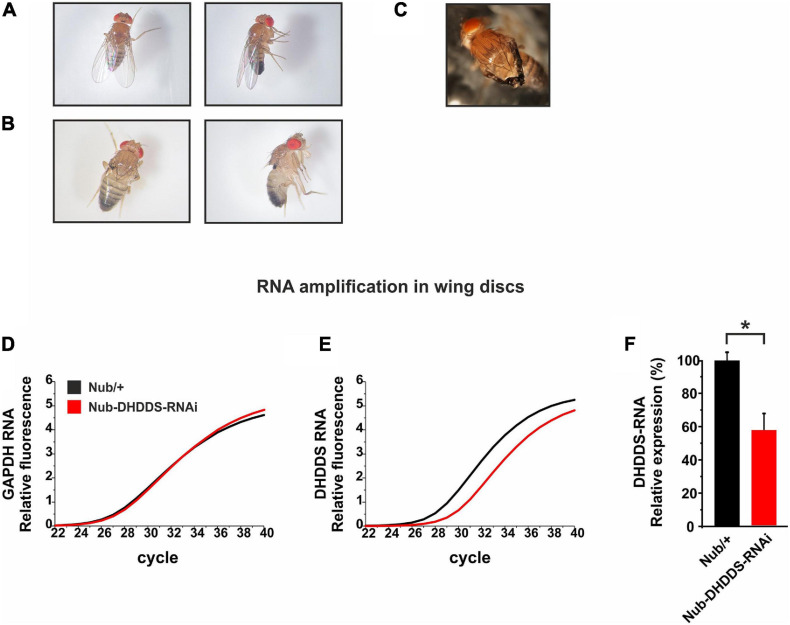
Knockdown of *DHDDS* in the developing wing primordium resulted in a complete wing loss. Nub-Gal4 flies were mated with either *w*^1118^ or UAS-DHDDS-RNAi flies (VDRC #104188). **(A,B)** Representative photographs taken under a stereo-microscope of the wings of F1 progenies, females (left) and males (right). **(A)** Nub/+ control flies. **(B)** Nub-DHDDS-RNAi flies showing wing absence, indicating that DHDDS is essential for wing formation. **(C)** A different UAS-DHDDS-RNAi line with a weaker phenotype. Nub-Gal4 flies were also mated with UAS-DHDDS-RNAi flies of a different line (VDRC #3166). The F1 progenies showed a weaker wing phenotype having twisted wings. **(D–E)** A graphical representation of the significant decrease in DHDDS mRNA levels following its RNAi-mediated knockdown in the wing imaginal discs. q-RT-PCR analysis of DHDDS mRNA expression levels in wing discs of control (Nub/+, black) and experimental (Nub-DHDDS-RNAi, red) third instar larvae. **(F)** Average expression of DHDDS mRNA in wing discs of experimental group (Nub-DHDDS-RNAi, red), relative to the control group (Nub/+, black). q-RT-PCR analysis was done using the Comparative threshold Cycle quantification that was used to calculate differential mRNA. Values were normalized to the control group. Statistical significance (*P ≤ 0.05) was determined using the Mann–Whitney *U* test in a one-tailed test. Data is presented as mean ± standard error of the mean (SEM), *n* = 3. The Mann–Whitney *U* test is a non-parametric test that allows two groups, conditions or treatments to be compared without assuming that values are normally distributed.

We used the following fly strains: *w*^1118^ (white-eyed fly serving as a control), w; *trp^*P*343^* (BDSC #9046), w; *ninaE^*I*17^* (BDSC #5701), *UAS-CG10778-RNAi* (VDRC #104188, #3166), *UAS-GFP* (BDSC #1522). The VDRC has confirmed that there are no off-targets to the CG10778-RNAi #104188. In our detailed study we used only the VDRC #104188 line because the #3166 line showed a weak phenotype (Figure 2C). The *CG10778-RNAi* was expressed using the Gal4/UAS binary system with the following drivers: white-eyed *GMR-Gal4*, [*GMR-Gal4*, UAS-*w*-RNAi] a gift from the lab of A. Huber, *nub*-Gal4 (BDSC # 25754), *act*-*Gal4/CyO* (BDSC #4414), and ninaE-Gal4.Ta (Rh1-Gal4) a gift from A. Huber. Flies were raised at 24°C in a 12h dark/light cycle.

### Western Blots Analysis

**FIGURE 3:**
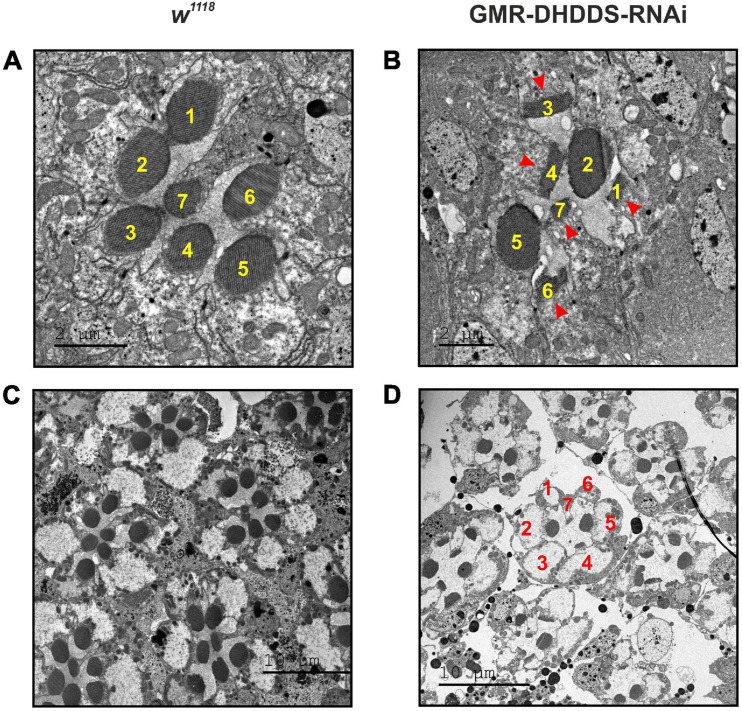
Retinal TEM sections of GMR-DHDDS-RNAi knockdown flies revealed a unique pattern of photoreceptors degeneration. Thin TEM sections at a ∼40 μm depth (from the corneal surface) of dark-raised newly eclosed *w*^1118^ **(A,C)** and experimental GMR-DHDDS-RNAi **(B,D)** retina at high magnification (×2000, **A** and ×1500, **B**) and low magnification (×500, **C,D**). The identity of the various photoreceptors was determined by the position of the easily identified R7. **(A)** A representative TEM section of a single ommatidium of a newly eclosed dark-raised w^1118^ fly in which the different photoreceptors are identified by their numbers. **(B)** A representative TEM section of a single ommatidium of a newly eclosed dark-raised GMR-DHDDS-RNAi fly in which the different photoreceptors are identified by their numbers. Note the normal appearance of the rhabdomeres of R2 and R5 cells and the degenerated appearance of the other cells showing very short microvilli (arrows). **(C)** Seven neighboring ommatidia are presented at a lower magnification (×500). Similar TEM with a normal retinal structure was previously described for the white-eyed GMR/+ control strain of older age (Weiss et al., 2012). Note the normal appearance of the rhabdomeres of **R2** and **R5** cells and the degenerated appearance of the other cells showing very short microvilli (arrows). **(D)** Several neighboring ommatidia of the GMR-DHDDS-RNAi fly are presented at lower magnification (×500).

To measure the levels of several *Drosophila* retinal signaling proteins, five one-day old fly heads or ten eyes were homogenized in a buffer solution and processed as previously described (Weiss et al., 2012). Following the protein transfer from the gel to the PVDF membrane, we dissected the membrane into three sections. Each section was probed with a different antibody: anti-Rh1 (monoclonal, 1:1,000 dilution), anti-TRP (polyclonal, 1:1000 dilution), and anti-dMoesin (polyclonal, 1:10,000 dilution; a gift from F. Payre). The density in each lane was corrected by the dMoesin signal serving as a loading control protein and calculated as a percentage of the control fly signals (Chorna-Ornan et al., 2005). The α-dMoesin antibody was used as a loading control protein because dMoesin resides at the base of the rhabdomeres (Chorna-Ornan et al., 2005) and therefore its level is only little affected upon ommatidial degeneration (see Figure 3 and Supplementary Figure 3).

### Electroretinogram and Light Stimulation

Electroretinogram (ERG) recordings were applied to intact flies as previously described (Peretz et al., 1994; Zhu, 2011; Weiss et al., 2012). The maximal luminous intensity at the eye surface was ∼3.5 logarithmic intensity units above the intensity for a half-maximal response. The prolonged depolarization afterpotential [PDA, (Minke, 2012)] was induced by a series of intense blue (B; Schott, BG 28 broad-band filter) light pulses and suppressed by orange (O; Schott OG 590 edge filter) light pulses (see Figure 7). This paradigm included two intense orange light pulses followed by an intense blue light pulse, which converted ∼80% of the Rh1 photopigment from the Rhodopsin (R) to the Metarhodopsin (M) pigment state in control flies (*w*^1118^) resulting in a prolonged depolarization (PDA) that continued in the dark long after the light was turned off. An additional intense blue light elicited small responses that originated from the central cells (R7-8) in which PDA was not induced, due to their UV-absorption spectra, whereas the R1–6 cells were non-responsive (inactivated) due to maximal activation of the channels as indicated by the disappearance of the “on” transient, which arises from activation of the second order lamina neurons by the R1-6 cells. The following orange light suppressed the PDA after light was turned off. The PDA was used as a convenient and reliable measure for the reduction of the Rh1 photopigment level *in vivo* (Minke, 2012).

### Transmission Electron Microscopy (TEM)

Flies were anesthetized on a CO_2_ pad and the eyes were removed separately, as fast as possible, by a scalpel and processed as previously described (Weiss et al., 2012). Briefly, white-eyed GMR-DHDDS-RNAi and *w*^1118^ flies were used. Fly heads were separated and bisected longitudinally from flies at two different ages: newly eclosed, and 12-days-old. Tissues including compound eyes were dissected out of the flies in fixative solution (5% glutaraldehyde, 0.1 M cacodylate buffer, pH 7.4) and incubated overnight. Samples were then post-fixed (1% OsO_4_, 0.1 M cacodylate buffer, pH = 7.4), dehydrated through a graded series of ethanol, and embedded in epoxy resin. Ultrathin sections stained with uranyl acetate and lead citrate were observed with a Tecnai-12 transmission electron microscope (FEI) and photographed with a Mega-view II charge-coupled device camera (Philips).

Since in *Drosophila* there are several mechanisms of light-dependent retinal degeneration, we wanted to exclude the possibility that the observed degeneration is due to a light-dependent mechanism. Therefore, we only used dark-raised flies in the present study.

### RNA Extraction and Quantitative Reverse Transcription PCR (q-RT-PCR)

Total RNA was isolated from either wing discs, eye discs or retinae of control (Nub/+ for wing discs and GMR/+ for eye discs and retinae) and experimental (Nub-DHDDS-RNAi for wing discs and GMR-DHDDS-RNAi for eye discs and retinae) groups. For wing and eye discs, late third instar larvae were dissected (50 for each sample). Briefly, wondering third instar larvae were collected and dissected in PBSx1 (an equal number of discs were taken from each group). For each wing disc the notum was removed, and the pouch (presumptive wing blade) was collected. Similarly, antenna discs were removed before the eye discs were collected. Discs were collected in the QIAGEN RNeasy mini kit’s lysis buffer and vortexed. Retinae were dissected from newly eclosed flies and put in tubes sitting on dry-ice. Total RNA was extracted by Trizol as described previously. To avoid any genomic DNA contamination, the DNase I Kit (Sigma, AMPD1-1KT) was used, according to the manufacturer’s instructions. cDNA was made using the High-Capacity cDNA Reverse Transcription Kit (Applied Biosystems, #4368814) containing RNase Inhibitor, using an equal amount of total RNA from each sample. q-RT-PCR analyses were done using a pre-optimized TaqMan Gene Expression Assay (Applied Biosystems) for measuring the relative expression of CG10778 (DHDDS; Dm01833749_g1; RefSeq: NM_132197.3; exon boundary: 1–2; and Assay Location 793) compared to the that of the reference gene, GAPDH (Dm01841185-m1; RefSeq NM_001273182.1; and exon 2-3 Assay Location 693). q-PCR was performed using a StepOne plus PCR instrument. Each reaction was performed in triplicate and the results were confirmed by three independent experiments. For each experiment, the CT values of the target gene in both groups were standardized by reference to the housekeeping gene, GAPDH (ΔCt). Then, the mean ΔCt of control group was calculated from all experiments, and used to calculate ΔΔCt values. The relative expressions were calculated by the 2^–ΔΔ*Ct*^ method. The relative expression of the target gene in the control group was set as 100%, and was normalized accordingly in the experimental group. Statistical significance (^∗^*P* ≤ 0.05) was determined using the Mann–Whitney *U* test, in a one-tailed test. Data is presented as mean ± standard error of the mean (SEM).

### Immunostaining of the Eye-Discs

Eye discs from late third instar larvae (in the range of 8 h prior to pupation) were fixed and stained using standard techniques (Callejo et al., 2011). The specific primary antibodies that were used: mouse anti-Elav (1:50; DSHB #9F8A9 supernatant). Rabbit anti Spalt (sal) (1:1000; gift from Adi Salzberg). Secondary antibodies that were used: Rhodamine Red-X anti mouse and cy5 anti rabbit from Jackson Labs at 1:400 dilution. Mounting medium containing Dapi was used (VECTASHIELD, #H1200). Images were captured by Nikon Ti2E confocal fluorescent microscope with Yokogawa W1 Spinning Disk integrated with 50 μm pinhole and lens ×20 or ×40. Figures were edited using Nis Elements software.

## Results

Human subjects with specific mutations in *DHDDS* (Figure 1) revealed non-syndromic RP (Zelinger et al., 2011; Züchner et al., 2011). To study the effect of compromising DHDDS on retinal structure and function in a model animal, we examined the consequences of targeted expression of *CG10778*-RNAi (DHDDS-RNAi) using the GMR-Gal4 driver and other tissue-specific promoters. *CG10778* is the ortholog of human *DHDDS*, (Figure 1). To this end, we crossed the *Drosophila* UAS-DHDDS-RNAi line with several Gal4 strains having different tissue-specific promoters that drive expression in various tissues at various developmental stages.

### Driving the DHDDS-RNAi to the Entire Body by the Actin Promoter

To study the consequence of compromising DHDDS expression throughout the fly embryo, UAS-DHDDS-RNAi flies were crossed with a Gal4 strain driven by the actin promoter (act-Gal4/CyO). Actin is a key component of the cytoskeleton ubiquitously expressed in all eukaryotic organisms. We found that all the resulting offspring (F1) exhibited the wing marker Curly (CyO), indicating that early ubiquitous knockdown of DHDDS is lethal. We conclude that DHDDS activity is vital during early fly development.

### Driving of the DHDDS-RNAi to the Developing Wing by the *nub* Promoter

We next examined the effect of DHDDS supression during the formation of specific tissues. The *Drosophila* imaginal wing disc of the larva gives rise to two adult derivatives: the notum (a trunk component) and the wing appendage. In order to examine the involvement of DHDDS in the formation of the wing, UAS-DHDDS-RNAi flies were crossed with the nubbin (Nub)-Gal4 strain, which drives expression selectively in the presumptive adult wing blade. The resulting F1 Nub-DHDDS-RNAi flies of the VDRC #104188 line revealed a complete wing loss in both female and male flies (Figures 2A,B), or partial wing loss when using the #3166 UAS-DHDDS-RNAi VDRC line which had a much weaker phenotype (small and twisted wings, Figure 2C). Therefore, we used the #104188 VDRC line for all our experiments. The results in Figure 2 clearly indicated that DHDDS is essential for wing formation.

To examined the efficiency of the RNAi-mediated knockdown of DHDDS, we dissected the wing imaginal discs of F1 Nub-DHDDS-RNAi late third instar larvae and determined the level of DHDDS mRNA using q-RT-PCR. For each wing disc the notum was removed, and the presumptive wing parts were collected for further analysis. We found a significant reduction (57.74 **±** 9.59%, SEM) in DHDDS mRNA levels relative to control Nub/+ (Figures 2D–F). This result clearly shows that the RNAi approach worked well to knockdown DHDDS expression.

### Driving of the DHDDS-RNAi to the Retina by the GMR Promoter

After elucidating the efficiency of DHDDS-RNAi in suppressing DHDDS expression (Figure 2), we applied a retinal-specific driver to target DHDDS-RNAi expression to the retina. We used the GMR-Gal4 driver, which is expressed in all cells posterior to the morphogenetic furrow in the developing eye (Song et al., 2000). The GMR promoter is the strongest and most commonly used driver for retinal expression. Although previous studies reported mild eye phenotypes associated with the GMR-Gal4 driver (Helms et al., 1999; Hiesinger et al., 1999; White and Jarman, 2000), the adverse phenotypes appear mainly in GMR-Gal4 homozygotes or when GMR-Gal4 flies are raised at 29°C, while GMR-Gal4 heterozygotes usually appear normal or have weak phenotype when raised at 25°C. In all of our experiments, we used only GMR-Gal4 heterozygotes raised at 24°C and used GMR-Gal4/+ (GMR/+) as a positive control to estimate the relative effect of possible phenotype. As measured by q-RT-PCR, targeted expression of DHDDS-RNAi using the GMR-Gal4 driver in the developing eye disc of the larva or in the retinae of newly eclosed GMR-DHDDS-RNAi flies slightly reduced DHDDS transcripts levels relative to the GMR/+ control. However, this reduction was not significant (88.74 **±** 13.65%, SEM in eye discs and 89.91 **±** 28.02%, SEM in newly eclosed retinae, Supplementary Figure 1). The GMR driver is expressed in the eye disc and at early pupa stages (Ray and Lakhotia, 2015). The fact that GMR-Gal4 expression in the eye disc is restricted, as it is not expressed in cells anterior to the morphogenetic furrow, may explain why a significant reduction of the DHDDS transcripts in the eye-disc was not detected. To visualize GMR-Gal4 expression in the developing eye we crossed GMR-Gal4 with UAS-GFP flies and collected late third instar larvae. Indeed, immunostaining of these eye-discs clearly showed that the area covered by the GMR-Gal4 is less than half of the entire eye disc (Supplementary Figure 2). According to the wing-disc experiments (Figure 2), DHDDS-RNAi reduced the level of the transcripts by no more than 40%. Therefore, the effect of GMR-Gal4 driven knockdown of DHDDS is expected to be mild in the entire eye-disc and hard to detect using q-RT-PCR, as was actually observed (Supplementary Figure 1).

### Targeted Expression of DHDDS-RNAi Resulted in a Unique Pattern of Retinal Degeneration

To assess the consequences of DHDDS-RNAi expression on adult retinal structure, we performed TEM on the retinae of dark-raised GMR-DHDDS-RNAi flies. Serial TEM thin retinal cross sections of newly eclosed flies, from the corneal surface down to ∼100 μm eye depth, revealed a unique pattern of ommatidial degeneration in GMR-DHDDS-RNAi flies compared to the normal retinal morphology of dark-raised white-eyed control flies (*w*^1118^, Figures 3A,C) or GMR/+ control flies of older age (Weiss et al., 2012). TEM of single ommatidia at high magnification (×1500) of GMR-DHDDS-RNAi flies revealed reduced rhabdomere area of most photoreceptor cells (Figure 3B). Strikingly, sections at a specific retinal depth revealed a non-uniform degenerative pattern of the signaling compartment (the rhabdomeres) within the ommatidia of GMR-DHDDS-RNAi flies. Interestingly, at a depth of ∼40 μm from the corneal surface the degree of photoreceptor degeneration and rhabdomeral cross section area appeared to be selective: the rhabdomeres of photoreceptors **R2** and **R5** appeared similar to control retinae, while all other rhabdomeres of the same ommatidia (i.e., R1, R3, R4, R6, and R7) at the same retinal depth did not show the typical round shape and revealed shorter microvilli than the control photoreceptors (Figures 3B,D). The above GMR-DHDDS-RNAi TEM sections (see also Supplementary Figure 3) are exceptional compared to previous TEM sections of mutant flies showing retinal degeneration (e.g., *rdgA, rdgB, rdgC, pp100*, and *cap*), which all exhibited a uniform pattern of degeneration of all R1-6 cells at different retinal depths (Iakhine et al., 2004; Leonard et al., 1992; O’Tousa et al., 1989; Rubinstein et al., 1989; Weiss et al., 2012). Interestingly, the study of the *cpn* transgenic fly (Weiss et al., 2012), demonstrated an RNAi-mediated suppression of the *cpn* gene-product, under the same GMR-Gal4 driver used in this study, causing retinal degeneration that lacked the unique pattern described in the present study.

**FIGURE 4:**
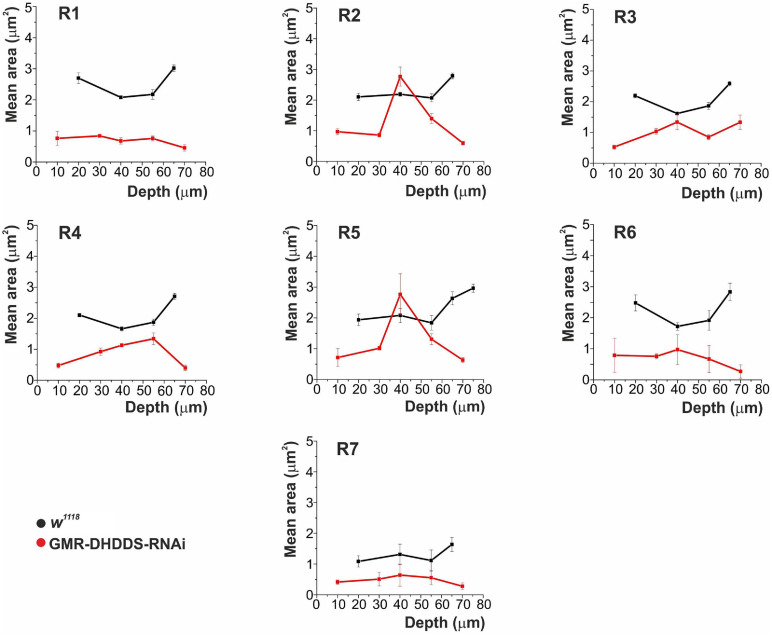
The mean cross-section area of rhabdomeric slices measured from R1-7 photoreceptors as a function of retinal depth. Analysis of serial TEM retinal cross sections of newly eclosed flies. Four sections were analyzed for the control group (*w*^1118^), at the depths of 20, 40, 55, and 65 μm from corneal surface. Six sections were analyzed for the experimental group (GMR-DHDDS-RNAi), at the depths of 10, 30, 40, 55, 70, and 85 μm from corneal surface. For each depth, 4–19 ommatidia were analyzed. The values in the graphs represent the mean area (in μm^2^) of every **R1–7** rhabdomere, as a function of depth, ±STDEV (Standart deviation). Measurements were done using the ImageJ and Origin softwares. A comparison was performed between the mean area of **R2** or **R5** at the retinal depth of 40 μm and the mean area of **R1**, serving as a representative rhabdomere from the group of R1, R3, R4, and R6, at the same retinal depth. While the mean area of **R2** and **R5** at 40 μm was 2.1 ± 0.4 and 2.4 ± 0.5 μm^2^, respectively, the mean area of R1 at the same depth was 0.6 ± 0.1 μm^2^ (average ± SEM, *n* = 6 for each group) and the differences between the groups were significant *p* ≤ 0.01 (**) for the difference between **R2** and **R1**, and *p* ≤ 0.05 for the difference between **R5** and **R1** (a two tailed, unpaired student’s *t*-test). At all other depths, **R2** and **R5** of GMR-DHDDS-RNAi retinal sections exhibited similar areas in comparison to **R1**, **R3**, **R4**, and **R6**. Note the smaller cross section area of all rhabdomeres of GMR-DHDDS-RNAi flies (red) relative to the *w*^1118^ control flies (black) at all measured retinal depth *except* for **R2** and **R5** rhabdomeres at ∼40 μm depth, where cross-section areas were similar to that of *w*^1118^ flies. The cell nucleus resides at ∼ 40 μm depth from the corneal surface.

To quantify the unusual pattern of degeneration, serial thin retinal TEM sections were analyzed for both control (*w*^1118^) and experimental (GMR-DHDDS-RNAi) flies. Sections at increasing depths of the retina (i.e., at 10, 20, 30, 40, 55, 65, 70, and 85 μm from corneal surface) were used to measure the area of all rhabdomeres as a function of depth (Figure 4). The results clearly show that in the depth of ∼40 μM from corneal surface the rhabdomeres of **R2** and **R5** cells exhibited a significantly larger cross section area (similar to that of the control *w*^1118^ flies) relative to all other rhabdomeres (i.e., R1, R3, R4, R6, and R7). This difference was quantified by a comparison between the mean area of **R2** or **R5** at the retinal depth of 40 μm and the mean area of R1 serving as a representative rhabdomere from the group of R1, R3, R4, and R6, at the same retinal depth. While the mean area of **R2** and **R5** at 40 μm was 2.1 **±** 0.4 μm^2^, and 2.4 **±** 0.5 μm^2^, respectively, the mean area of R1 at the same depth was 0.6 **±** 0.1 μm^2^ (average **±** SEM, *n* = 6 for each group) and the differences between the groups were significant (see caption of Figure 4). At all other depths, R2 and R5 of GMR-DHDDS-RNAi retinal sections exhibited similar areas in comparison to R1, R3, R4, and R6.

**FIGURE 5:**
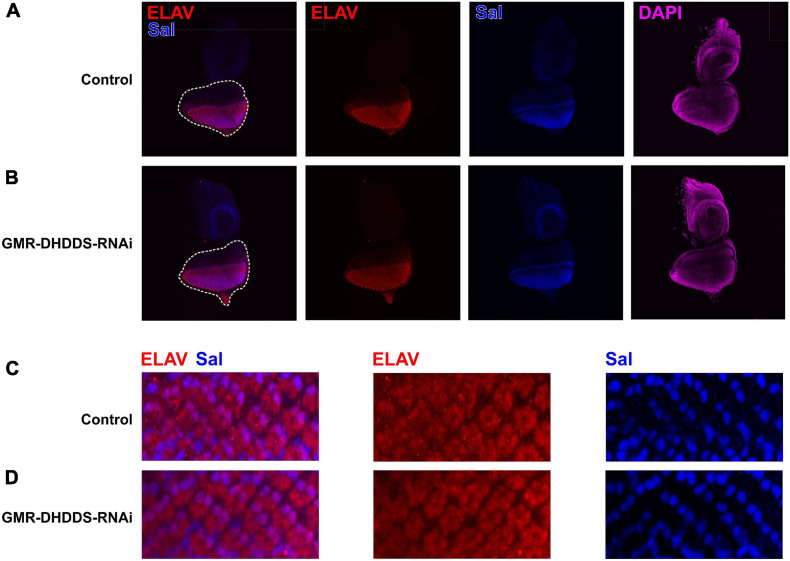
Immunostaining of the GMR-DHDDS-RNAi imaginal eye discs. Eye discs of GMR-DHDDS-RNAi and control (GMR/GFP) late third instar larvae were collected and stained with DAPI (nuclei, magenta), ELAV (developing photoreceptors, red), and Sal (R3/R4 differentiation and planar polarity, blue). **(A,B)** ×20 magnification of control GMR/GFP **(A)** and GMR-DHDDS-RNAi **(B)** imaginal eye discs. **(C,D)** ×40 magnification of sections of control **(C)** and GMR-DHDDS-RNAi **(D)** imaginal eye discs. The sections were selected to include Sal expression at R3/R4. Note that ELAV and Sal are normally expressed in the GMR-DHDDS-RNAi and control tissues.

To determine whether the observed retinal phenotype is due to an early larval defect, we analyzed the GMR-DHDDS-RNAi eye discs. Staining for the neuronal marker Elav revealed a normal differentiation pattern of photoreceptors similar to that seen in the control eye discs (Figure 5). The transcription factor Spalt (Sal) is required for R3/R4 specification and planar cell polarity establishment (Domingos et al., 2004b). In addition Sal promotes terminal R8 differentiation during pupal stages, including the regulation of rhodopsin expression (Domingos et al., 2004a). Co-staining for Sal showed that control GMR/+ and GMR-DHDDS-RNAi eye discs are indistinguishable (Figure 5), indicating that DHDDS knockdown is not interfering with normal larval eye disc development. These results indicate that the GMR-DHDDS-RNAi expression exerted its effect during the pupal stage, possibly when the rhabdomeres differentiate to form the adult retina.

**FIGURE 6:**
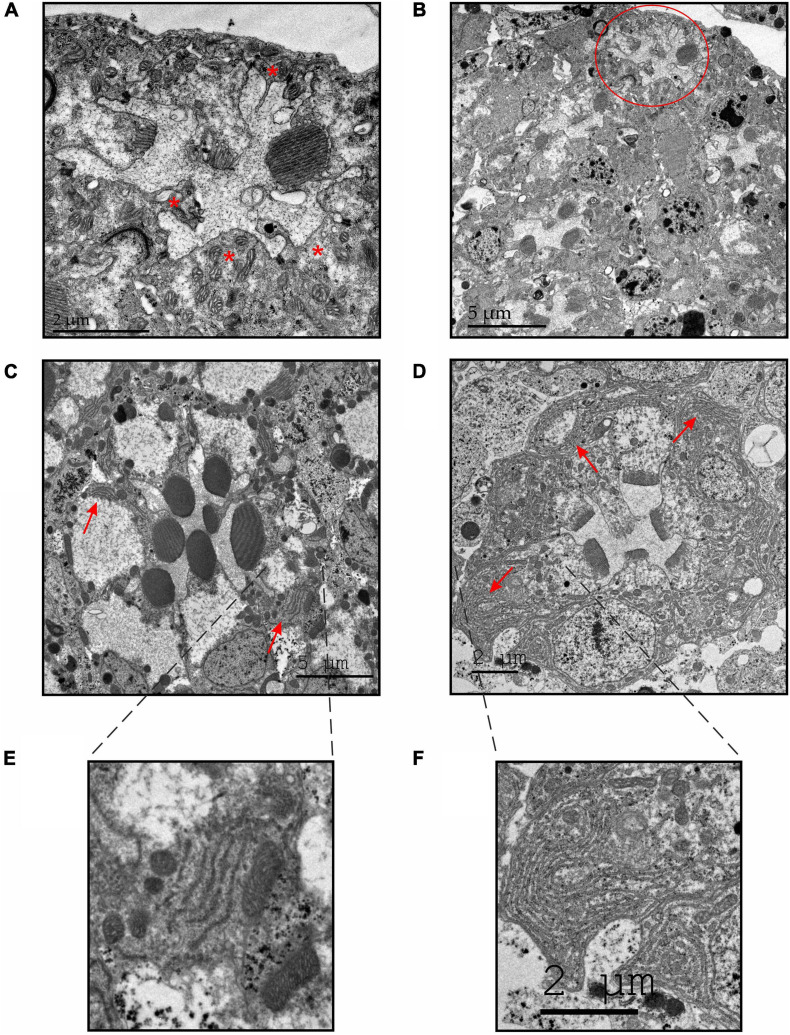
TEM sections of GMR-DHDDS-RNAi flies revealed an enhanced degeneration with increasing age and an abnormally large accumulation of ER membranes in the affected retinae. **(A,B)** Representative thin TEM cross retinal sections at a ∼55 μm depth (from the corneal surface) showing an ultrastructure of a 12-days-old GMR-DHDDS-RNAi fly at a high (×2500, **A**) and low (×800, **B**) magnification. Missing rhabdomeres are marked with asterisks in **(A)**. The ommatidium shown in **(A)** is marked with a red circle in **(B)**. **(C,D)** Representative thin TEM cross retinal sections at a ∼55 μm depth (from the corneal surface) of dark-raised newly eclosed control (*w*^1118^) flies **(C)** and GMR-DHDDS-RNAi flies **(D)** at a high magnification. The red arrows show the normal appearance of ER membranes in **(C)** and the abnormal accumulation of ER membranes in **(D)**. **(E)** An enlarged image of the indicated area in **(C)** showing a normal manifestation of ER membranes in the control (*w*^1118^) ommatidium. **(F)** An enlarged image of the indicated area in **(D)** showing an abnormal accumulation of ER membranes in the affected retinae of GMR-DHDDS-RNAi fly.

To further determine whether the deformed appearance of the rhabdomeres deteriorate with age, we examined the retinal ultrastructure of 12-days-old GMR-DHDDS-RNAi flies. We used TEM cross sections at high (×2500, Figure 6A) and low (×800) magnification (Figure 6B). Importantly, these older retinae exhibited a more advanced stage of degeneration accompanied by the loss of two or more rhabdomeres in each ommatidium indicating cumulative degeneration with age.

### Accumulation of ER Membranes in the Degenerating GMR-DHDDS-RNAi Photoreceptors

The mechanism underlying photoreceptor degeneration of the GMR-DHDDS-RNAi flies is unknown. Nevertheless, a clearly abnormal accumulation of ER-membranes was observed in newly eclosed GMR-DHDDS-RNAi relative to *w*^1118^ retinae (Figures 6C,D red arrows, Figures 6E,F) or white-eyed GMR/+ control flies of older age (Weiss et al., 2012). Interestingly, it was found that in the *Drosophila* rhodopsin mutant Rh1N20I, which lacks the single N-glycosylation site, a severe defect in rhodopsin maturation was accompanied by massive accumulations of ER membranes (Colley et al., 1991; O’Tousa, 1992; Baker et al., 1994; Colley et al., 1995). This accumulation of ER membranes was a general consequence of improper rhodopsin processing during maturation (Colley et al., 1991; Baker et al., 1994; Colley et al., 1995) resulting in low rhodopsin protein levels in the photoreceptors (O’Tousa, 1992). The abnormal accumulation of ER membranes in the GMR-DHDDS-RNAi flies (Figure 6D red arrows, Figure 6F) suggests an impairment of Rh1-N-glycosylation accompanied by a severe reduction in Rh1 rhodopsin level (Katanosaka et al., 1998).

### A Reduction in the Prolonged Depolarizing After Potential Signal in GMR-DHDDS-RNAi Flies

**FIGURE 7:**
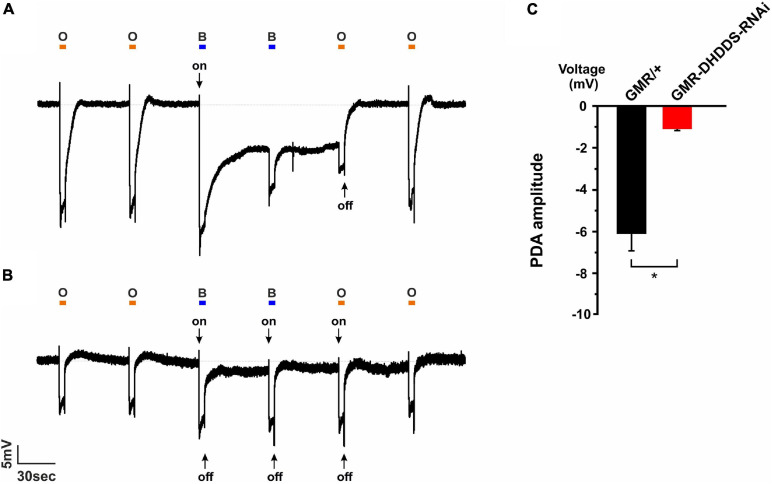
Prolonged Depolarizing Afterpotential (PDA) revealed a highly reduced Rh1 rhodopsin level in GMR-DHDDS-RNAi flies in vivo. **(A)** A representative ERG response to intense blue and orange lights of a 21-days-old white-eyed control (GMR/+) fly. A similar ERG response was observed in a newly eclosed fly of the same strain, indicating the preservation of the structure and function of the control flies (not shown). The orange bars represent orange light pulses (O; Schott OG 590 edge filter), while the blue bars represent blue light pulses (B; Schott, BG 28 broad-band filter). The first blue light pulse induced a PDA in R1–6 photoreceptors by converting Rh1 rhodopsin to its dark stable state, metarhodopsin. An on transient is indicated (arrow). The following blue light-pulse elicited neither PDA nor on-and-off transients, but only an ERG response of the R7, 8 cells. An additional orange light-pulse converted metarhodopsin back to Rh1 rhodopsin and suppressed the PDA at the cessation of the light. The following orange light did not convert a net amount of photopigment and did not elicit a PDA. **(B)** An ERG response of a newly eclosed white-eyed GMR-DHDDS-RNAi fly showed a very short PDA, on-and-off transients (see arrows) and a smaller ERG response amplitude under an identical experimental paradigm, indicating a highly reduced Rh1 rhodopsin level. **(C)** A histogram comparing the PDA amplitude of 21-days-old white-eyed GMR/+ (black) and newly eclosed white-eyed GMR-DHDDS-RNAi flies (red). The baseline was set to 0mV, and the voltage was measured 55 seconds after the first blue light was turned off. Data is presented as mean ± standard error of the mean (SEM), *n* = 11. The difference between the two group was significant (*p* ≤ 0.05, a two-tailed, unpaired student’s *t*-test).

To examine whether the accumulation of ER membranes in the GMR-DHDDS-RNAi flies is also accompanied by a reduction in Rh1 rhodopsin level, we first used a reliable and sensitive physiological assay. This assay indirectly measures the reduction in Rh1 level in the retina by an electroretinogram (ERG) recording of the Prolonged Depolarizing After potential (PDA). The PDA that is measured by ERG recording is an *in vivo* electrophysiological signal with amplitude and duration that depend on the Rh1 photopigment level (Minke, 2012). In our experiments, we used 21 days-old control (GMR/+) flies and newly eclosed GMR-DHDDS-RNAi flies in order to emphasize the quality of our control. Since the GMR promoter is sometimes associated with small structural deformations effects that can be worsened with age (Helms et al., 1999; Hiesinger et al., 1999; White and Jarman, 2000), it was important to show that a normal control PDA is preserved even at the “old” age of 21 days. Thus, the strong PDA reduction in the newly eclosed GMR-DHDD-RNAi flies did not arise from properties of the GMR/+ background. The PDA represents the summed electrical activity of the eye in white-eyed flies, in response to a specific paradigm of illumination: two consecutive intense blue light pulses, followed by an intense orange light pulse. The PDA of *Drosophila* is induced after the first light pulse, when intense blue light converts a net large amount of the Rh1 rhodopsin to its dark-stable intermediate state, metarhodopsin. Accordingly, intense blue light applied to a 21-days-old control white-eyed fly (GMR/+) induced a robust light response in the R1-6 photoreceptor cells that failed to terminate upon the cessation of the light stimulus and generated a continuous saturated depolarization in the dark. The second intense blue light induced a smaller response superimposed on the PDA that arises from R7, R8 cells in which PDA was not induced (Figure 7A). The input of the R1–R6 cells in the second order neurons is revealed in the ERG response by the production of on- and off-transients (Figure 7A, arrows). When Rh1 rhodopsin level is normal and R1-6 cells enter a full PDA, their PDA response in the dark is saturated and no on- and off-transients appear in the response to the second blue light (Figure 7A). The PDA was suppressed by the following intense orange light that converted metarhodopsin back to rhodopsin (Figure 7A). The amplitude and duration of the PDA critically depend on the amount of Rh1 photopigment conversion, which is determined by the photopigment level. A reduction of the photopigment level in the fly results in reduced PDA amplitude and duration and the appearance of on- and off-transients in the response to the second blue light [Figure 7B, (Minke, 2012)]. Application of the PDA protocol to eyes of newly eclosed GMR-DHDDS-RNAi flies showed low PDA amplitude (Figures 7B,C), short PDA duration and appearance of on-and -off transients at the beginning and end of all light responses (Figure 7B). Taken together, the PDA phenotype of the GMR-DHDDS-RNAi flies indicates a highly reduced Rh1 level.

### A Reduction in the Protein Levels of Rh1 Rhodopsin in GMR-DHDDS-RNAi Flies

**FIGURE 8:**
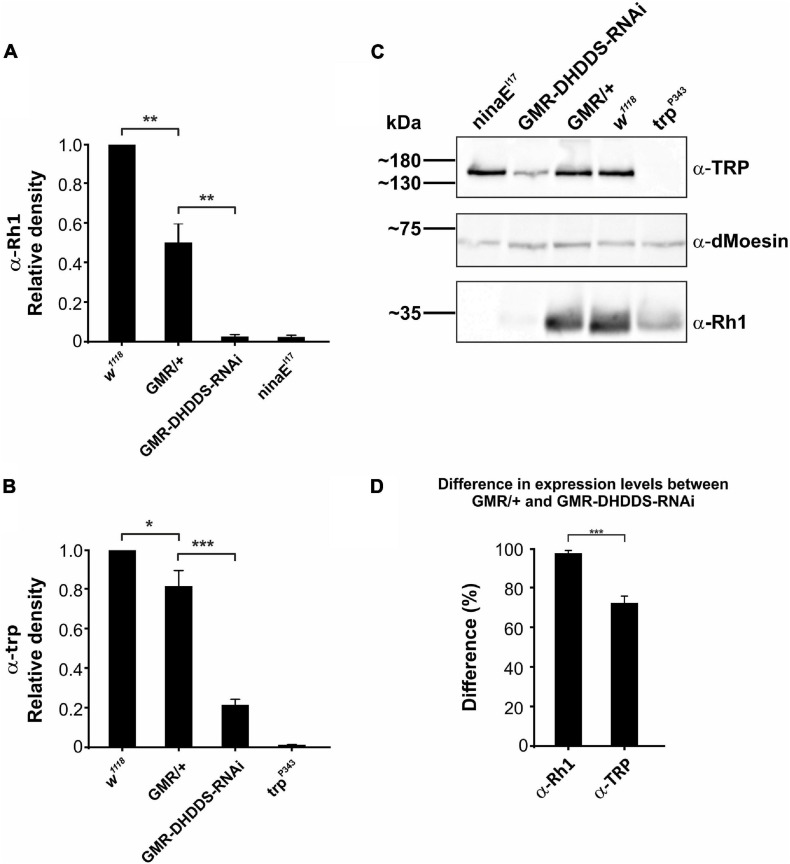
A drastic reduction of Rh1 rhodopsin levels in GMR-DHDDS-RNAi flies. Western blot analysis that compares the expression levels of Rh1 rhodopsin and TRP channel of newly eclosed white-eyed GMR-DHDDS-RNAi fly relative to positive control of newly eclosed white-eyed *w*^1118^ control. **(A)** A histogram showing a drastic reduction of Rh1 rhodopsin level in GMR-DHDDS-RNAi flies relative to positive control. The first and second columns show the Rh1 level of w^1118^ and GMR/+, while the third column shows the Rh1 level of GMR-DHDDS-RNAi flies. The 4th column shows a negative control of the *ninaE^*I*17^* Rh1 null mutant. For each group, the histogram shows the average value of normalized αRh1 denseties from all experiments, relative to the control (*w*^1118^) group. Statistical analysis was done using the Student’s *t*-test, in a two-tailed paired test. Data is presented as mean ± standard error of the mean (SEM), *n* = 6 or 7 for each group. A significant (*P ≤ 0.05) difference in αRh1 densities was found between the GMR/+ and *w*^1118^ groups, and between the GMR-DHDDS-RNAi and the GMR/+ groups. **(B)** A histogram showing a reduction of TRP channel protein level in GMR-DHDDS-RNAi flies relative to the positive control. The first and second columns show TRP level of *w*^1118^ and GMR/+ of the same flies used in section **(A)**, while the third column shows the TRP level of GMR-DHDDS-RNAi flies. The 4th column shows a negative control of the *trp^*P*343^* a TRP null mutant. For each group, the histogram shows the average value of normalized α-TRP denseties from all experiments, relative to the control (*w*^1118^) group. Statistical analysis was done using the Student’s t-test, in a two-tailed paired test. Data is presented as mean ± standard error of the mean (SEM), *n* = 6 or 7 for each group. A significant (*P ≤ 0.05) difference in α-TRP densities was found between the GMR/WT and WT groups, and between the GMR-DHDDS-RNAi and GMR/WT groups (***P ≤ 0.001). **(C)** A representative Western blot showing an example of a blot used in the histograms. Heads or eyes were lysed and run on gel. Following the protein transfer from the gel to a PVDF membrane, we dissected the membrane into three sections. Each section was probed with a different antibody: αRh1 (monoclonal, 1:1,000 dilution), αTRP (polyclonal, 1:1000 dilution), and α-dMoesin (polyclonal, 1:10,000 dilution). The density of the α-Rh1 and α-TRP bands were corrected by the dMoesin signal serving as a protein loading control, and calculated as a percentage of control fly (*w*^1118^) signals. **(D)** A histogram showing the difference between Rh1 rhodopsin and TRP channel protein levels in the GMR-DHDDS-RNAi flies, and their levels in the GMR/+ flies. For each target protein (Rh1 rhodopsin or TRP channel) the mean difference between expression levels in the GMR/+ flies and expression levels in the GMR-DHDDS-RNAi flies was calculated. In each gel (*n* = 6), the expression level of each target protein (Rh1 rhodopsin or TRP channel) in the GMR/+ flies was set as 100%, from which the expression level of the same protein in the GMR-DHDDS-RNAi was subtracted. The mean difference between Rh1 rhodopsin levels in the GMR/+ flies and Rh1 rhodopsin levels in the GMR-DHDDS-RNAi flies was 97.2 ± 1.2% (SEM). The mean difference between the TRP protein levels in the GMR/+ flies and the mean difference of the GMR/+ was 72.1 ± 3.3% (SEM). A significant (***P ≤ 0.001, a two tailed, unpaired student’s *t*-test) difference was found between the mean difference of Rh1 rhodopsin levels and the mean difference of the TRP channel protein levels.

To measure the Rh1 levels directly, we applied Western blot analysis using a monoclonal Rh1 antibody. White-eyed GMR-DHDDS-RNAi flies (Figure 8C, lane 2, from the left) were used to examine the change in Rh1 expression, while white-eyed GMR/+ and *w*^1118^ flies (Figure 8C, lanes 3, 4) were used as positive controls and the white-eyed *ninaE* Rh1 null mutant (*w; ninaE^*I*17^*) was used as a negative control (Figure 8C, lane 1). The Western blot analysis and the accompanied histogram that quantitated the data revealed a drastic reduction in the expression levels of Rh1 in GMR-DHDDS-RNAi flies, relative to the positive controls (Figures 8A,C). Since the GMR-DHDDS-RNAi flies exhibited retinal degeneration, we examined whether the drastic reduction in Rh1 levels was solely due to this degeneration, or whether a part of it could be attributed to the DHDDS-RNAi expression. To answer this question, we used TRP as a marker for rhabdomeral deformations. The TRP channel of *Drosophila* uses the same secretory pathway as Rh1 to reach the surface membrane and both are major signaling membrane proteins of the photoreceptors that reside in the microvilli (Rosenbaum et al., 2011). The TEM data showed that already at eclosion most rhabdomeres, where both TRP and Rh1 reside, are abnormally small (Figure 3 and Supplementary Figure 3). Therefore, a reduction in both TRP and Rh1 was expected. We measured the levels of the TRP channel protein (relative to its control) in the same experimental groups of flies, and used white-eyed *trp* null mutant (*w*; *trp^*P*343^*) as a negative control (Figures 8B,C, lane 5 from the left). The histogram (Figure 8D) presents the relative difference between each signaling protein (Rh1 or TRP) expression level in the control group (GMR/+) and its corresponding level in the GMR-DHDDS-RNAi group. A 72.1 ± 3.3% (SEM) reduction was observed in the expression level of the TRP channel protein in the GMR-DHDDS-RNAi flies relative to the TRP level in control flies, while the reduction in the level of Rh1 protein was 97.2 ± 1.2% (SEM) in the GMR-DHDDS-RNAi flies relative to the Rh1 level in control flies.

We interpreted these results as follows: the reduction in the TRP protein level reflects the partial degeneration of the rhabdomeres, where both TRP and Rh1 reside. The larger relative reduction in the Rh1 protein level reflects both the rhabdomeres’ partial degeneration and a suppression of Rh1expression caused by the DHDDS-RNAi expression. This is in accordance with the pivotal role of DHDDS in N-glycosylation, which is essential for Rh1synthesis (Kumar et al., 1997). We attribute the observed decrease in Rh1 and TRP expression levels in the GMR/+ control flies, compared to that of the *w*^1118^ flies, to the previously reported, mild degenerative effects associated with the GMR-Gal4 driver (Helms et al., 1999; Hiesinger et al., 1999; White and Jarman, 2000). Nevertheless, the large and highly significant reduction of both Rh1 and TRP protein levels in the GMR-DHDDS-RNAi flies relative to the GMR/+ control flies fully justified the use of this strong promoter.

## Discussion

Mutations in the human DHDDS have been associated with RP, a disease resulting in retinal degeneration and hence blindness. Here we investigated the role of *Drosophila* DHDDS enzyme in the fly retina, using RNAi-mediated knockdown, in order to identify cellular and molecular mechanisms, which might be involved in this retinal disease. Early ubiquitous knockdown of DHDDS resulted in lethality, while targeted knockdown in the larval wing blade primordium led to wing loss. A likely explanation for these results, based on the fact that the product of DHDDS enzymatic activity is dolichol, is that compromising DHDDS activity should result in impaired N-glycosylation of essential developmental proteins along the fly developmental stages.

Targeted expression of DHDDS-RNAi using an eye-specific Gal4 driver (GMR-Gal4) resulted in several defects in the organization and function of the ommatidia. These phenotypes were manifested in a defective rhabdomere structure including shorter or occasionally no microvilli, expanded ER and a very strong reduction in the amount of Rh1 protein in addition to a reduction of the ERG response to light of the photoreceptor cells. The following observation supports the notion that these adverse phenotypes change over time. Retinal TEM sections of newly eclosed GMR-DHDDS-RNAi flies when compared to 12-days-old flies reveled a worsened with age retinal degeneration. In contrast, GMR/+ control flies of similar age did not show ommatidial degeneration (Weiss et al., 2012). These results exemplify the cumulative nature of the retinal degeneration associated with compromised DHDDS expression.

One of the striking observations of this study is the highly unusual pattern of retinal degeneration in GMR-DHDDS-RNAi flies that showed a reduced degeneration of **R2** and **R5** rhabdomeres at the region of their nucleus in the newly eclosed GMR-DHDDS-RNAi flies. In none of the known retinal degeneration *Drosophila* mutants [e.g., *rdgA* (Inoue et al., 1989), *rdgB* (Rubinstein et al., 1989), *rdgC* (Steele and O’Tousa, 1990), *pp10*0 (Iakhine et al., 2004), and *cpn* (Weiss et al., 2012)] there is a similar pattern of degeneration. Elucidating the developmental mechanism underlying this unique pattern of retinal degeneration is beyond the scope of this work. However, unlike the differences in the pattern of degeneration between vertebrate rods and cones, the asymmetric retinal degeneration GMR-DHDDS-RNAi flies is not due to differential expression of photopigments, as R1-6 cells express Rh1 rhodopsin, and these cells do not express any other photopigments. Thus, it is reasonable to assume that it is related to the relationship between DHDDS-dependent processes and properties that distinguish **R2** and **R5** cells from the other photoreceptors. Notably, among the R1-R7 cells, **R2** and **R5** cells are the first to be specified as photoreceptors. Moreover, a mechanism that discriminates **R2** and **R5** from the other photoreceptors was found in a study of the *rough* gene which encodes for a homeobox transcription factor that is expressed in the early developing eye only in photoreceptors **R2** and **R5** (Tomlinson et al., 1988). Strikingly, *rough* is not required for **R2** and **R5** differentiation, rather, it is required, non-autonomously, for the specification of the other photoreceptors that are subsequently added to the developing ommatidia. It is therefore possible that either the early specification time of **R2** and **R5** cells or their distinctive genetics underlies their differential response to the impairment of DHDDS enzymatic activity induced in GMR-DHDDS-RNAi flies.

The product of DHDDS enzymatic activity is dolichol, which is essential for N-glycosylation (Webel et al., 2000). Studies using *Drosophila* found that N-glycosylation of rhodopsin protein occurs in the ER and is required for its maturation, but not for rhabdomeric membrane assembly or for its role in the phototransduction cascade. Mutation of the rhodopsin N-glycosylation site leads to retention of the immature protein within the secretory pathway, causing an accumulation of ER membranes and low rhodopsin protein levels (O’Tousa, 1992; Webel et al., 2000). Accordingly, the drastic reduction of Rh1 level and ER membranes accumulation observed following retinal knockdown of DHDDS expression is consistent with a reduction in Rh1 N-glycosylation as the mechanism underlying the observed retinal degeneration. Mammalian TRPC (i.e., TRPC6 and TRPC3) channels also undergo N-glycosylation, but the observed retinal degeneration is not due to impairment of TRP N-glycosylation since mutation-induced removal of its glycosylation site had virtually no effect on the level of the TRP channel protein, but only made the channel constitutively active (Dietrich et al., 2003). Interestingly, the glycosylation site of mammalian TRPC channels is not conserved in the *Drosophila* TRP channel. Moreover, an extensive survey of membrane-associated glycoproteins and their N-glycosylation sites isolated from the adult wild-type *Drosophila* fly heads did not identify the photoreceptor TRP channel among the a total of 205 glycoproteins carrying N-linked glycans (Koles et al., 2007). Our results support a model in which targeted expression of DHDDS-RNAi resulted in a unique pattern of retinal degeneration and a drastic reduction of Rh1 rhodopsin level. Since this is the first presentation of a *Drosophila* model useful for studying the *Drosophila* ortholog of the mammalian DHDDS, functional roles of this important protein remain to be determined in future studies.

A recent study in mice retina, in which dolichol production was suppressed, revealed a profound retinal degeneration with no evidence for defective protein N-glycosylation in this mouse model, despite confirmed ablation of DHDDS in the entire population of retinal rod cells (Ramachandra Rao et al., 2020). It is possible that the photoreceptor degeneration observed in our study might not be related to alteration in opsin *N*-glycosylation, but rather to a toxic accumulation of isoprenoid compounds produced during dolichol biosynthesis. The reduced dolichols level may also affect other cellular functions by influencing membrane fluidity.

## Data Availability Statement

The datasets presented in this study can be found in online repositories. The names of the repository/repositories and accession number(s) can be found in the article/Supplementary Material.

## Author Contributions

TBr performed most of the electrophysiological experiments, part of the Western blot experiments, and most of the q-RT-PCR experiments, participated in the TEM experiments, analyzed the data, prepared the final figures, and drafted parts of the manuscript. RI performed genetic parts of the study, parts of the electrophysiological, and Western blot experiments, analyzed and quantitated the TEM data. TBi designed and performed parts of the genetic study, dissected the imaginal discs, performed the immunostaining and confocal imaging of eye discs, and contributed to the writing of the manuscript. RZ performed part of the q-RT-PCR and participated in the Western blot analysis. LM-M performed the initial preliminary experiments. DS supervised the initial experiments and suggested the strategy of the study. VK established the initial results in flies and suggested the strategy of the study. OG designed and supervised the genetic part of the study, interpreted the data, drafted and wrote the manuscript. BM designed and supervised the entire project, interpreted the data, drafted and wrote the manuscript. All authors contributed to the article and approved the submitted version.

## Conflict of Interest

The authors declare that the research was conducted in the absence of any commercial or financial relationships that could be construed as a potential conflict of interest.

## Supplementary Material

Supplementary Figure 1 Targeted suppression of *DHDDS* expression in the developing eye disc of the larva or in the retinae of newly eclosed flies did not reduce *DHDDS* transcripts levels. **(A,B)**. A graphical representation of the amplification of GAPDH **(A)** vs. DHDDS **(B)** mRNA, extracted from eye discs of both control group (GMR/+, black) and experimental group (GMR-DHDDS-RNAi, red). The amplification curves of the target gene DHDDS are similar in both groups. The experimental protocol was identical to that of Figure 2. **(C)** Average expression of DHDDS mRNA in eye discs of experimental group (GMR-DHDDS-RNAi, red), relative to the control group (GMR/+, black). q-RT-PCR analysis was done using the Comparative threshold cycle (CT) quantification method that was used to calculate differential mRNA levels (see Figure 2). Values were normalized to the control group. Statistical analysis was done using the Mann–Whitney *U* test, in a one-tailed test. Data is presented as mean ± standard error of the mean (SEM), *n* = 4. No significant reduction in the mRNA of the target gene DHDDS was found in the experimental group, relative to the control group. **(D,E)** A graphical representation of the amplification of GAPDH **(D)** vs. DHDDS **(E)** mRNA, extracted from retinae of both control group (GMR/+, black) and experimental group (GMR-DHDDS-RNAi, red). The amplification curves of the target gene DHDDS are similar in both groups. **(F)** Average expression of DHDDS mRNA in retinae of experimental group (GMR-DHDDS-RNAi, red), relative to the control group (GMR/+, black). q-RT-PCR analysis was done using the Comparative threshold Cycle quantification that was used to calculate differential mRNA. Values were normalized to the control group. Statistical analysis was done using the Mann–Whitney *U* test, in a one-tailed test. Data is presented as mean ± standard error of the mean (SEM), *n* = 3. No significant reduction in the mRNA of the target gene DHDDS was found in the experimental group, relative to the control group. The GMR driver is expressed in the eye disc and at early pupa stages (Ray and Lakhotia, 2015). To explain why a significant reduction of the target transcript of DHDDS was not detected **(C)**, we refer to Supplementary Figure 2 showing that GMR/GFP is only expressed in the morphogenetic furrow and posterior to it, namely, in a limited volume of the eye disc (Supplementary Figure 2), unlike the situation in the wing disc (Rodríguez et al., 2002). Since we took for the analysis the entire eye-disc, the DHDDS-RNAi was diluted with non-relevant RNA and therefore DHDDS transcript reduction could not be significantly detected. To account for the lack of a significant reduction of the target transcript of DHDDS in the newly eclosed isolated retinae **(E)**, we note that in the isolated retinae of the newly eclosed transgenic fly, the q-RT-PCR of DHDDS and GMR/+ retinae showed a rise in fluorescence only after 29.9 cycles, indicating a minute level of DHDDS transcript in both (**E**, see also Figure 2E, red curve showing a rise in fluorescence after 28.8 cycles, for a comparison). The minute levels of DHDDS transcript prevented an observation of a significant difference between GMR/+ and GMR-DHDDS-RNAi transcripts in the isolated retinae.

Supplementary Figure 2 Immunostaining of the *Drosophila* imaginal eye discs. Eye discs of GMR-Gal4/GFP (GMR/GFP) late third instar larvae were collected and stained with DAPI (that stained the nuclei, blue), and ELAV (that stained the developing photoreceptors, red). GFP (green) labels the expression of the GMR promoter. **(A)** *Merged localization of DAPI, GFP*, and *ELAV*. The area of the eye disc is circled with a dashed line. Note that the expression of the GMR promoter (marked by GFP) is restricted to the region posterior to the morphogenetic furrow, covering only a half of the eye disc region. **(B) *Localization of all the nuclei*,** in the entire eye disc and antenna section. **(C) *Localization of the expression region of the GMR promoter.*** Note that the expression of the GMR promoter is restricted to the region posterior of the morphogenetic furrow. **(D)** *Localization of the developing photoreceptors*. Note that the expression of the developing photoreceptors is restricted to the region posterior of the morphogenetic furrow. **(E)** *Merged localization of DAPI and GFP*.

Supplementary Figure 3 Retinal TEM section of GMR-DHDDS-RNAi knockdown flies at low magnification showing a pattern of retinal degeneration in the retina. A representative low magnification (×300) TEM cross section at a ∼55 μm depth (from the corneal surface), showing an ultrastructure of a newly eclosed GMR-DHDDS-RNAi fly. Note that the typical organized ommatidial structure is preserved (see dotted lines). In addition, most of the rhabdomeres are present, with a degenerated appearance.
