# Supplementary figures and images for: Knockdown of Dehydrodolichyl Diphosphate Synthase in the Drosophila Retina Leads to a Unique Pattern of Retinal Degeneration
Source: Front Mol Neurosci. 2021 Jul 5;14:693967. doi: 10.3389/fnmol.2021.693967 (PMC8287061; doi:10.3389/fnmol.2021.693967)

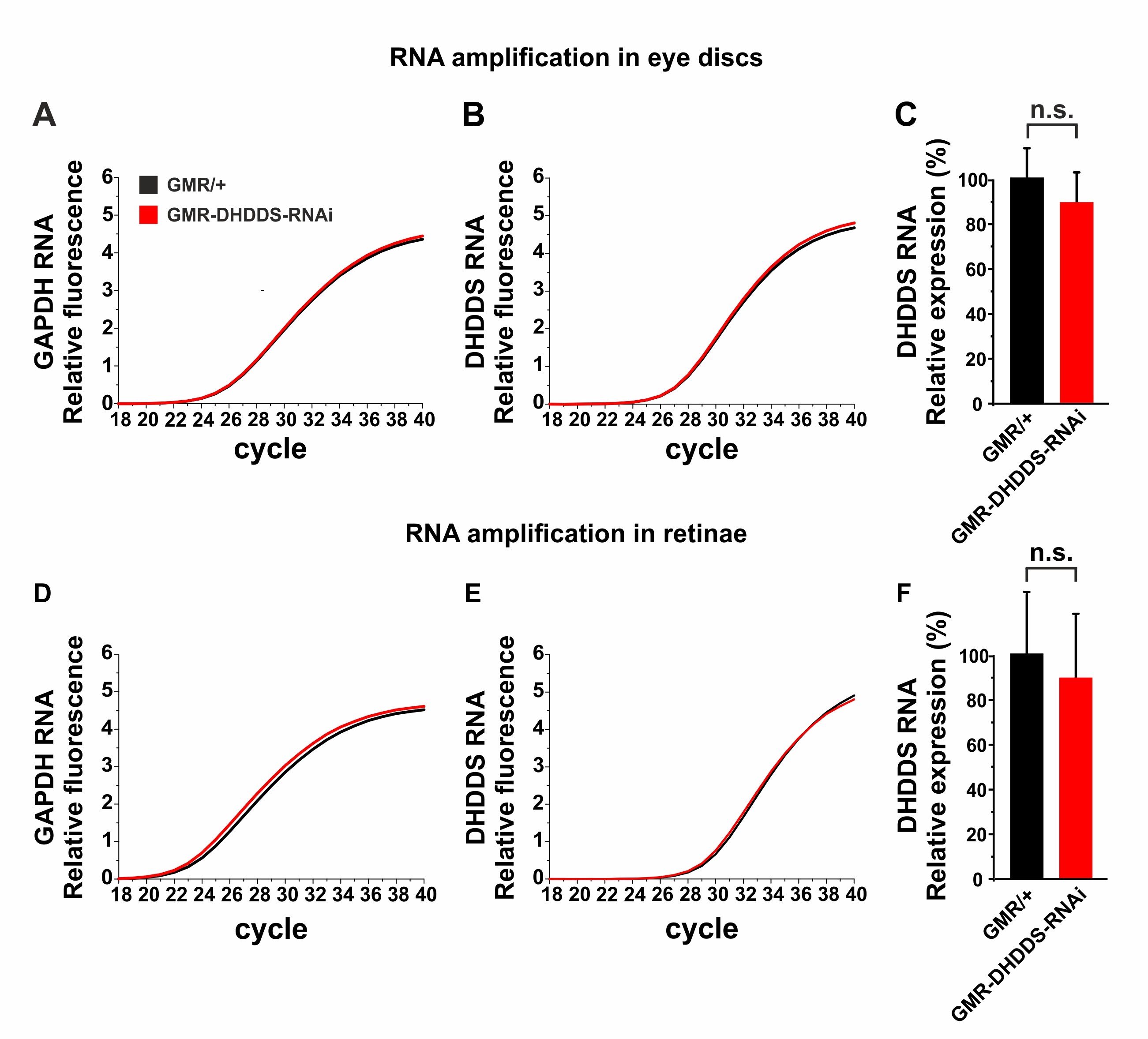

Supplement: Supplementary Figure 1 — Targeted suppression of DHDDS expression in the developing eye disc of the larva or in the retinae of newly eclosed flies did not reduce DHDDS transcripts levels. (A,B). A graphical representation of the amplification of GAPDH (A) vs. DHDDS (B) mRNA, extracted from eye discs of both control group (GMR/+, black) and experimental group (GMR-DHDDS-RNAi, red). The amplification curves of the target gene DHDDS are similar in both groups. The experimental protocol was identical to that of Figure 2. (C) Average expression of DHDDS mRNA in eye discs of experimental group (GMR-DHDDS-RNAi, red), relative to the control group (GMR/+, black). q-RT-PCR analysis was done using the Comparative threshold cycle (CT) quantification method that was used to calculate differential mRNA levels (see Figure 2). Values were normalized to the control group. Statistical analysis was done using the Mann–Whitney U test, in a one-tailed test. Data is presented as mean ± standard error of the mean (SEM), n = 4. No significant reduction in the mRNA of the target gene DHDDS was found in the experimental group, relative to the control group. (D,E) A graphical representation of the amplification of GAPDH (D) vs. DHDDS (E) mRNA, extracted from retinae of both control group (GMR/+, black) and experimental group (GMR-DHDDS-RNAi, red). The amplification curves of the target gene DHDDS are similar in both groups. (F) Average expression of DHDDS mRNA in retinae of experimental group (GMR-DHDDS-RNAi, red), relative to the control group (GMR/+, black). q-RT-PCR analysis was done using the Comparative threshold Cycle quantification that was used to calculate differential mRNA. Values were normalized to the control group. Statistical analysis was done using the Mann–Whitney U test, in a one-tailed test. Data is presented as mean ± standard error of the mean (SEM), n = 3. No significant reduction in the mRNA of the target gene DHDDS was found in the experimental group, relative to the control group. The GMR dri [file Image_1.JPEG]

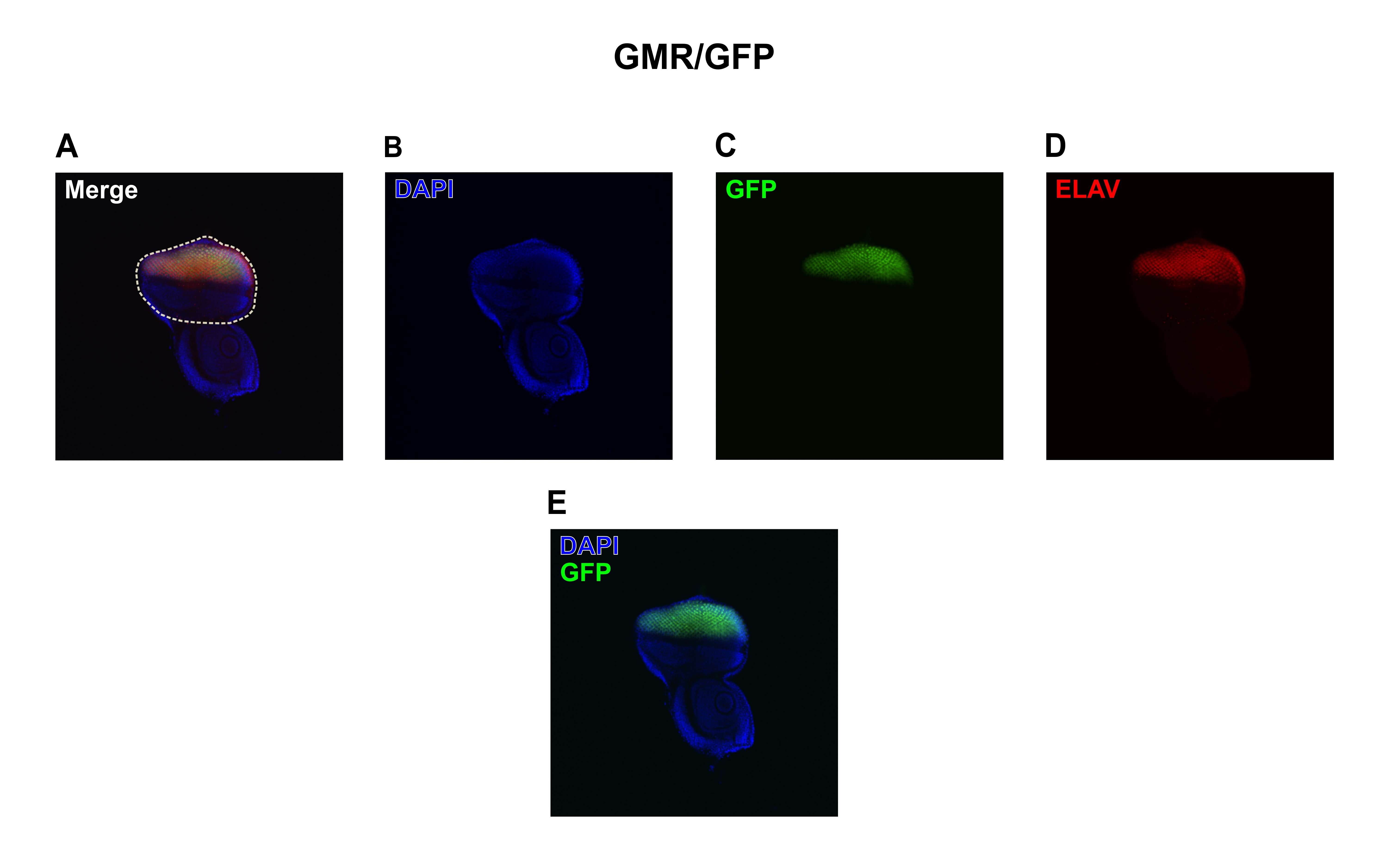

Supplement: Supplementary Figure 2 — Immunostaining of the Drosophila imaginal eye discs. Eye discs of GMR-Gal4/GFP (GMR/GFP) late third instar larvae were collected and stained with DAPI (that stained the nuclei, blue), and ELAV (that stained the developing photoreceptors, red). GFP (green) labels the expression of the GMR promoter. (A) Merged localization of DAPI, GFP, and ELAV. The area of the eye disc is circled with a dashed line. Note that the expression of the GMR promoter (marked by GFP) is restricted to the region posterior to the morphogenetic furrow, covering only a half of the eye disc region. (B) Localization of all the nuclei, in the entire eye disc and antenna section. (C) Localization of the expression region of the GMR promoter. Note that the expression of the GMR promoter is restricted to the region posterior of the morphogenetic furrow. (D) Localization of the developing photoreceptors. Note that the expression of the developing photoreceptors is restricted to the region posterior of the morphogenetic furrow. (E) Merged localization of DAPI and GFP. [file Image_2.JPEG]
